# Supplementary material for: Are carbon emissions trading and green financial instruments synergistic? -Comprehensive quantitative research based on content analysis
Source: PLoS One. 2024 Mar 7;19(3):e0298601. doi: 10.1371/journal.pone.0298601 (PMC10919601; doi:10.1371/journal.pone.0298601)
Supplement: S1 Data — (ZIP) [file pone.0298601.s001.zip › Data/地级市Z_F工作报告.zip!/║╙─╧/┬σ╤⌠╩╨/└┤╫╘╣1⁄2╓┌║┼í╛┬φ┐╦╩2╛▌═°í┐_2018.docx]

各位代表：

现在，我代表市人民政府，向大会报告工作，请予审议，并请各位政协委员和其他列席人士提出意见。

一、本届政府工作回顾

本届政府履职以来、特别是“十三五”以来，在市委的坚强领导下，全市人民以习近平新时代中国特色社会主义思想为指导，深入学习贯彻习近平总书记调研指导河南时的重要讲话精神，认真落实市第十一次党代会及历次全会精神，紧盯“四高一强一率先”奋斗目标，围绕理念落地构建体系、围绕体系推进谋划专项、围绕专项实施抓好项目，统筹抓好稳增长、促改革、调结构、惠民生、防风险各项工作，加快推进“9+2”工作布局落地见效，保持了经济社会持续健康发展的良好态势，中原城市群副中心城市地位不断彰显，全省增长极的支撑作用更加突出。

经济持续平稳增长。全市生产总值由2013年的3140.8亿元增加到2017年的4343.1亿元、年均增长8.8%，高于全省0.5个百分点，经济总量占全省比重回升至9.7%。规模以上工业增加值年均增长9.6%，高于全省0.7个百分点。固定资产投资年均增长16%，高于全省1.1个百分点。一般公共预算收入年均增长10.3%。截至2018年8月，全市金融机构人民币各项存款余额5557.8亿元、贷款余额3873.1亿元，分别是2013年末的1.66倍和1.97倍。

大事要事取得突破。自创区、自贸区、“中国制造2025”等国家战略先后落地，示范带动效应初步显现。我市相继入选国家电子商务示范城市、电子功能材料区域集聚试点、小微企业创业创新基地城市示范、循环经济示范城市等重大试点，为长远发展赢得了更多的政策支持。省委、省政府出台《关于支持洛阳市加快中原城市群副中心城市建设的若干意见》，在洛设立15亿元历史文化遗址保护发展专项资金和500亿元军民融合产业投资基金。呼南高铁豫西通道路线方案获得国家铁路总公司认可。地铁1、2号线快速推进，洛阳正在迈向“地铁的新时代”。投资110亿元的洛阳石化炼油结构调整一期工程、投资88.8亿元的洛宁抽水蓄能电站、投资44.6亿元的前坪水库等重大项目开工建设，分别投资150亿元的格力自主创新智能制造产业基地、银隆新能源产城融合产业园全面加快实施。

动能转换步伐加快。大力实施“三大改造”，规划建设高端装备制造等六大重点产业园，17个百亿级特色产业集群加快形成。“双倍增”行动成效显著，新增创新主体3680家、创新平台1167个；其中，省级创新龙头企业20家、高新技术企业169家，国家级创新平台29个，国家级专业化众创空间建设走在全国前列。新建院士工作站17个，引进“两院”院士、“千人计划”等创新人才（团队）21个。“聚力产业创新院士洛阳行”活动取得圆满成功。连续三届成功承办中国创新创业大赛先进制造行业总决赛。2017年，高新技术产业增加值占规模以上工业的比重较2014年提高8.8个百分点；服务业增加值增长10.8%，连续3年高于GDP和第二产业增速，对经济增长的贡献率达55.8%。“五强六新五特”现代产业体系、现代创新体系加快构建，洛阳产业结构实现了从“二三一”向“三二一”的历史性转变。

文化传承创新体系日益完善。丝绸之路、大运河双申遗成功。牡丹文化节、河洛文化旅游节持续提升，首届世界古都论坛、首届中原文化旅游产业博览会圆满成功。大遗址保护展示创造洛阳模式，二里头夏都遗址博物馆全面开工，汉魏洛阳城国家考古遗址公园基本建成，隋唐洛阳城国家历史文化公园、丝绸之路文化交流中心等项目加快推进。“博物馆之都”建设迈出坚实步伐，定鼎门遗址博物馆等一批各具特色的博物馆建成开放，博物馆总数达到68家。龙门石窟荣获联合国世界旅游组织公共政策管理创新奖。我市入选“中国文化遗产保护传承十佳城市”、国家公共文化服务体系示范区、全国“志愿之城”试点城市，连续3届蝉联“全国文明城市”。

城镇化水平不断提高。大力实施“一中心六组团”城市发展战略，着力形成“四级联动”城镇总体布局，现代城镇体系加快构建。国家新型城镇化综合试点建设任务圆满完成，全市城镇化率年均提高约1.65个百分点。古城快速路、滨河南路、新街跨洛河大桥、九都路东延长线、东环路、南环路等71项重点路桥工程建成通车，城市路网更加完善，“洛阳速度”和“保通先行”的做法受到群众好评。备受瞩目的城市规划馆竣工投用，成为展示洛阳形象的会客厅。兴洛湖公园、定鼎门广场、伊水游园八里堂段建成开放，132个四季有绿、功能齐全的小游园在市区遍地开花，为群众提供了健身、娱乐、休闲的好去处。提升改造背街小巷298条，打通断头路30条。美丽乡村建设稳步推进。

基础能力持续提升。加大交通、水利等重大工程建设力度，现代基础设施体系日益完善。6条高速公路同时在建，建成后通车总里程将提高66.5%，“三横三纵三环”高速路网加快形成。三洋铁路洛阳段开工建设。洛阳机场改扩建二期基本完工，国际航线正式开通，通航城市增至26个。引黄入洛、故县水库引水工程全线贯通。220千伏变电站、城市数字化管理平台实现县域全覆盖。新增集中供热面积3100万平方米，中心城区集中供热率从43%大幅提升至77.4%，惠及群众30万户。

改革开放全面深化。现代市场体系、现代开放体系日趋完善。供给侧结构性改革深入推进，国资国企、财税金融等重点领域改革取得阶段性成效。“放管服”改革成效明显，市级行政审批和中介服务事项分别精简55.5%和68%，非行政许可审批全面取消，营商环境持续改善，创新创业蔚然成风，各类市场主体达到39.3万家。自贸区省内首家线下国际贸易“单一窗口”建成投用，通关时间缩短近70%，累计入驻企业4000多家。引进省外境内资金3288.9亿元，实际利用外资120.7亿美元，入驻境内、境外500强企业分别达到27家和47家。

生态环境不断改善。环境保护意识显著增强，生态环境建设体系初步形成。PM10、PM2.5和空气质量优良天数实现“双下降、一上升”，人民群众的蓝天获得感进一步提升。建立四级河长体系，“四河同治、三渠联动”全面启动，完成河道治理139.4公里、排污口截流393处，整治黑臭水体18条，市县级水源地保护区综合整治、重点流域水污染防治等工作扎实推进，水环境质量实现稳定达标。我市入选国家清洁取暖试点城市，未来三年将获得国家9亿元资金支持。加大生态惠民力度，实施主要廊道沿线绿化提升27万亩，新建改造公园绿地669.8万平方米。

人民生活水平持续提高。全市各级财政民生支出累计1444.7亿元，年均增长12.7%。2017年，城乡居民收入分别是2013年的1.34倍和1.43倍。新增城镇就业57.91万人、失业人员再就业16.29万人、就业困难人员实现就业58.07万人。31万棚户区居民住上保障房。30万农村贫困人口实现脱贫，329个贫困村退出贫困序列，贫困发生率由2013年底的9.1%下降到3.84%。偃师市、洛龙区、伊滨经开区、高新区整体脱贫。易地扶贫搬迁工作名列全省前茅。农村低保标准与扶贫标准“两线合一”、同步提高。新建、改扩建公办幼儿园及农村小学附属学前班577所（个）、义务教育阶段学校1431所，其中农村寄宿制学校197所。“全面改薄”完成校舍类、装备类学校1327所。市中心医院、妇女儿童医疗保健中心恢复公益属性，提升乡镇卫生院68所。新增养老床位11442张。实现企业养老保险省级统筹和城镇居民医疗保险、城镇职工医疗保险、工伤保险、失业保险市级统筹。我市入选全国首批医养结合试点城市、居家和社区养老服务改革试点。建成投用城市书房36个，“15分钟阅读圈”正在形成。13.5万户偏远地区群众看上数字广播电视。国防动员和后备力量建设取得新进步，驻洛部队参与脱贫攻坚成效明显，军政、军民团结进一步加强，我市荣膺全国双拥模范城“七连冠”。

2017年，我们主要做了以下工作：

（一）精准施策稳增长，经济运行保持稳中向好。坚持稳中求进、多措并举、精准发力，2017年，全市生产总值同比增长8.7%。发展质量明显提高。50户重点工业企业利润同比增长1.5倍，带动全市规模以上企业利税、利润总额分别达到571.1亿元和351.4亿元，增长31%和50%，高于全省25和41.5个百分点；工业利润率上升至4.5%、为2012年以来最高。完成一般公共预算收入325.9亿元、增长10.8%。项目建设成果丰硕。大力推进“9643”合理有效投资行动计划，428个省市重点项目完成投资1176亿元、是年度目标的1.4倍。310国道改建、中信重工高端装备制造产业园二期等项目相继竣工，洛阳动车所、洛阳机场三期改扩建等项目前期工作取得实质性进展，与解放军信息工程大学合作的工业CT产业化项目积极推进。“牡丹号”盾构机正式下线。地企合作持续深化。累计签约产业项目235个，已开工221个；签约产销对接协议49个，协议金额25.85亿元，已履约25.97亿元；签约科技成果转化项目123个，已实施113个；签约产融合作协议271个，已履约571.36亿元，履约率77.8%。金融服务能力不断提升。银企洽谈会签约资金1411亿元、落实率91%。新增贷款462.4亿元，新增存贷比137.5%，创2012年以来新高。还贷周转金周转量达86亿元，直接减少企业融资成本2亿元以上。

（二）加快转型调结构，产业优化升级亮点纷呈。坚持创新驱动发展。高起点编制自创区发展规划纲要，确立“一核四区”空间布局，自创区核心区与省直部门直通车制度初步建立。出台激发大院大所大企业创新活力“金十条”，深挖“创新富矿”。一拖集团牵头组建的河南省智能农机创新中心入选省级制造业创新中心。中硅高科荣获中国专利金奖，洛阳石化工程公司荣获国家科技进步一等奖。洛阳科技金融大市场投入运行。全年专利申请量突破万件，技术合同成交额同比增长32.6%。军民融合稳步推进。成立中原军民融合产业联盟，设立军民融合“三基金一银行”，建设军民融合特色专业园区6个，谋划实施军民融合项目80个，军民融合产业园首批15个项目集中开工。制造业加速向中高端迈进。轴研科技、一拖集团、麦斯克公司入选国家智能制造应用项目。中船重工725所荣获全国质量奖。六大高成长性制造业对规上工业增长的贡献率达60.7%。现代服务业实现提质增效。全市接待游客1.24亿人次，旅游总收入突破千亿元，同比分别增长8.6%和15.3%。国际互联网数据专用通道开通试运行。信息服务业营业收入增长32.1%。广发银行、恒丰银行和8家证券公司相继落户；设立基金6支，新增上市（挂牌）再融资企业15家、中原股权交易中心挂牌企业140家。农业产业化步伐加快。新建高标准粮田4.95万亩，发展特色种植176万亩，粮食总产45亿斤。培育沟域经济示范区23个。我市入选全国休闲农业和乡村旅游示范市。孟津县入选全国休闲农业和乡村旅游十强县。

（三）深化改革促开放，发展动力活力持续增强。突出供给侧结构性改革主线，推动重点改革精准落地。“去降补”取得阶段性成效，去除煤炭产能435万吨；16家“僵尸企业”得到妥善处置；商品房库存去化周期下降至9.7个月；减轻企业负担40亿元；水利、科技服务、交通邮政等领域投资分别增长142.6%、82.1%和30.7%。资本市场融资406.1亿元。城投集团等6家企业子公司完成混合所有制改革；46家驻洛央企签订“三供一业”分离移交协议，29家全面实施；8家驻洛省企剥离企业办社会职能，56家市出资企业基本完成脱钩改制，国企内部“三项制度”改革有序推进。企业登记“三十五证合一”、全程电子化全面实施。城管综合执法体制改革基本完成。农村承包土地经营权确权登记颁证工作全省领先。文化、林业、公务用车、机关事业单位养老等领域改革成效明显。推广PPP模式和老工业基地改造受到国务院通报表彰。强化自贸区龙头带动，双向开放步伐加快。自贸区160项改革创新任务全面推进，洛阳跨境电商通关平台实现“一站式”通关。中储综合物流园、一拖多式联运物流中心项目有序推进。中亚国际货运班列顺利开通。国宏融资租赁公司获批运行。精准招商力度加大，全年签约亿元以上项目429个、投资总额2916.2亿元，开工率73.9%，其中10亿元以上项目80个、开工34个。全年实际利用外资近27亿美元、省外境内资金765.6亿元。完成外贸进出口总额133亿元。

（四）统筹城乡促协调，综合承载能力不断增强。城乡规划体系更加完善。启动城市五期总规修编和全市域空间试点规划编制工作，“一中心六组团”空间规划、县域新一轮规划、中心镇（特色镇）规划、26项专项规划编制和四期总规评估顺利完成。重大基础设施项目加快实施。洛阳至吉利新建黄河大桥双向通车，环城高速6个出入口改造大头落地。北环路、新伊大街等项目有序推进，伊滨中心城区道路环线全面贯通。生活垃圾焚烧发电厂建成投用，餐厨废弃物处理工程加紧实施。百城建设提质工程全面推进，405个项目加快实施，铺设市政管网431公里，9县（市）城中村、旧城改造累计新开工1179万平方米、竣工833万平方米。农村生产生活条件持续改善。新建农村公路777公里，新安县入选全国首批“四好农村路”示范县。农村危房改造超额完成年度目标。77个建制村完成环境综合整治。6.45万人饮水安全问题得到解决。节水灌溉、水土流失治理稳步推进。新增国家级、省级电子商务进农村综合示范县7个。嵩县入选全国农村幸福社区建设示范县。污染防治攻坚战初战告捷。整治取缔“散乱污”企业8814家，取缔燃煤散烧设施13119个，完成“气代煤、电代煤”7.2万户。全年空气质量优良天数达到210天。栾川县入选首批国家生态文明建设示范县。

（五）惠民利民增福祉，群众生活质量持续提升。加大民生投入，全年民生支出422.3亿元，占一般公共预算支出的76.9%。民生实事全面完成。脱贫攻坚成效显著。投入扶贫资金36.3亿元、增长68.4%。建成扶贫产业基地1732个、光伏扶贫电站218兆瓦，实施到户增收和科技扶贫项目362个、基础设施项目1421个，18368名贫困群众喜迁新居。公共服务体系日益完善。新增城镇就业12.27万人、农村劳动力转移就业3.05万人；发放创业担保贷款27亿元，支持1.9万人成功创业、带动6.8万人就业。降低保险费率，减轻企业、职工负担6.6亿元。市广播电视中心建成投用，市中心图书馆、文化馆、少儿图书馆、非遗中心等项目加快推进。市十三届运动会成功举办。市民之家建成投用，群众办事更加便捷。环城高速免费通行、牡丹文化节市民免费赏花等惠民政策赢得群众点赞。社会大局保持稳定。村委会、社区居委会换届选举顺利完成。持续开展安全生产专项整治，完成尾矿库综合治理122座，实现了“一杜绝、三下降”目标。食品药品安全形势平稳向好。积极防范化解重大风险，健全社会治安防控网络，加强社会治理创新，畅通群众诉求表达渠道，社会大局总体稳定。

（六）改进作风提效能，政府自身建设不断加强。认真学习贯彻习近平新时代中国特色社会主义思想和党的十九大精神，扎实推进“两学一做”学习教育常态化制度化，深入开展“不忘初心、牢记使命”主题教育，增强“四个意识”，坚定“四个自信”，自觉落实“两个坚决维护”。取消证明事项196项、减少94.7%，388项市级行政审批及服务事项的承诺办结时限比法定时限压缩60%以上。加强政府立法，法治政府评估连续三年居全省第一，依法行政工作被省政府通报表彰。自觉接受人大法律监督和工作监督，主动接受政协民主监督，办理人大代表建议293件、政协提案353件。正风肃纪力度进一步加大，“四查四促”专项行动共查处违反中央八项规定精神问题357个，处理553人。同时，民族宗教、外事侨务、对台、统计、审计、人防、气象、地震、史志、残疾人工作、妇女儿童事业等都取得了新的成绩。

今年以来，全市上下深入贯彻落实市第十一次党代会及历次全会和市委经济工作会议精神，坚持“四个着力”，发挥优势打好“四张牌”，坚决打好“三大攻坚战”，经济社会发展保持稳中有进、稳中向好的态势。上半年，全市实现生产总值2159.3亿元、增长8.8%，增速分别高于全国、全省2个和1个百分点，保持在全省第一方阵。

各位代表！近年来洛阳发展取得的成绩，是习近平新时代中国特色社会主义思想领航指路的结果，是以习近平同志为核心的党中央坚强领导的结果，是省委、省政府关心厚爱的结果，是市委站位全局、正确领导、科学决策的结果，是市人大、市政协加强监督、鼎力支持的结果，更是全市人民凝心聚力、务实重干的结果。在此，我代表市人民政府，向全市人民，向人大代表、政协委员，向各民主党派、人民团体，向离退休老领导、老同志，向驻洛部队、武警官兵和公安干警，向所有关心支持洛阳发展的社会各界和海内外朋友，表示衷心的感谢并致以崇高的敬意！

在肯定成绩的同时，我们也要清醒地看到，全市经济社会发展中还存在不少问题：初加工、高能耗产业比重仍然较大，新兴产业增长较快但支撑能力尚显不够，产业结构调整任务艰巨；创新引领发展的能力有待提升，新旧动能转换仍需加速；开放型经济发展水平不高，民营经济实力不强；城乡二元矛盾依然突出，县域经济支撑能力不强；防范化解重大风险、精准脱贫、污染防治三大攻坚战任务艰巨；民生领域欠账较多，就业、教育、医疗、养老不均衡等问题尚未得到很好地解决；政府职能转变不够到位，营商环境需进一步优化。对这些问题，我们一定高度重视，采取积极有效措施，认真加以解决，让全市人民感受到实实在在的变化和成效。

二、今后五年的目标任务

党的十九大确定了新的宏伟目标，波澜壮阔的新时代图景正在徐徐展开。未来五年是落实十九大精神的关键期，也是决胜全面小康的攻坚期，任务艰巨，使命光荣。

新时代开启了新的发展征程，我们要高举旗帜、牢记嘱托，在新思想指引下砥砺前行，向党和人民交出一份合格答卷。习近平新时代中国特色社会主义思想为我们实现“中国梦”指明了方向。“十三五”以来洛阳发展取得的成就，充分证明“9+2”工作布局、“四高一强一率先”奋斗目标符合中央精神和省委省政府要求，符合洛阳实际和人民意愿。只要我们保持定力，沿着正确的方向一抓到底、久久为功，习近平新时代中国特色社会主义思想就一定会在河洛大地落地生根、开花结果！

新时代带来了新的历史机遇，我们要乘势而上、勇于担当，扛起时代赋予的光荣使命，谱写中原更加出彩的洛阳篇章。自创区、自贸区和“中国制造2025”等国家战略在洛阳集中布局；省委、省政府赋予洛阳新的发展定位，量身打造了“真金白银”的支持政策，要求我们“跳出洛阳看洛阳、跳出河南看洛阳”，打好具有洛阳特色的“四张牌”。我们有信心、有决心，积极应对稳中有变新问题新挑战，抢抓机遇奋力实现新担当新作为，建好副中心、打造增长极，在中原更加出彩进程中“画”上浓墨重彩的一笔！

新时代承载着新的民生期盼，我们要团结拼搏、开拓进取，撸起袖子加油干，让洛阳人民对美好生活的向往变成现实。让老百姓过上好日子是我们一切工作的出发点和落脚点。只要我们始终坚持以人民为中心的发展思想，时刻把群众的安危冷暖放在心头，扎实办好群众最关心最期盼的民生实事，一件接着一件办、一年接着一年干，就一定能够把洛阳建设得更加美好，让古都人民的日子越过越红火。

今后五年，政府工作的总体要求是：以习近平新时代中国特色社会主义思想为指导，深入贯彻党的十九大精神、习近平总书记调研指导河南时的重要讲话精神、省委十届六次全会暨省委工作会议、市第十一次党代会及历次全会精神，紧扣我国社会主要矛盾变化，坚持新发展理念，坚持稳中求进工作总基调，坚持高质量发展的根本方向，统筹推进“五位一体”总体布局和协调推进“四个全面”战略布局，认真落实“四个着力”、打好“四张牌”、县域治理“三起来”、乡镇工作“三结合”等重要要求，以供给侧结构性改革为主线，统筹做好稳增长、促改革、调结构、惠民生、防风险各项工作，聚焦决胜全面小康、开启现代化建设新征程，聚焦加快建设副中心、打造全省增长极，聚焦全面深化“9+2”工作布局、奋力实现“四高一强一率先”奋斗目标，坚决打好防范化解重大风险、精准脱贫、污染防治的攻坚战，着力深化改革开放创新，抓重点、补短板、强弱项，推动经济发展质量变革、效率变革、动力变革，促进经济社会持续健康发展，在全省率先全面建成小康社会，朝着建设富强民主文明和谐美丽的现代化强市迈进，在中原更加出彩中谱写浓墨重彩的洛阳篇章。

今后五年政府工作的主要奋斗目标是：

综合实力再上新台阶。全市生产总值年均增长8%左右，增速高于全省平均水平；科技实力、创新能力和城镇化水平大幅跃升，研发投入占GDP的比重达到3%以上、科技进步贡献率达到65%以上；中原城市群副中心城市地位更加巩固，成为全省经济发展新的增长极，为全面建设社会主义现代化强市奠定坚实基础。

发展质量显著提升。财政收入占生产总值的比重高于全省平均水平，税收占财政收入比重不低于70%；产业高端化、绿色化、智能化、融合化水平明显提升，高新技术产业增加值占工业的比重达到45%；“9+2”工作布局全面深化落实，现代化经济体系建设取得明显成效，高质量发展格局基本形成。

城乡发展更加协调。新型城镇化质量明显提高，中心城市功能品位显著提升，县域经济支撑能力明显增强，乡村振兴取得重要进展，组团发展、四级联动、城乡一体的格局全面形成；常住人口城镇化率达到65%以上，户籍人口城镇化率达到50%以上，现代城镇体系基本确立。

生态环境明显改观。大气污染治理取得决定性成效，到2022年，城市区空气质量优良天数达到260天以上；水环境质量根本改善，土壤环境质量逐步好转，森林覆盖率45%以上；单位生产总值能耗下降16%，生产生活方式实现绿色化低碳化，资源环境承载能力显著提高，河洛大地天更蓝、地更绿、水更清、环境更优美。

社会治理更加高效。劳动就业、公共教育、医疗卫生、公共文化、社会保障等方面实现较大发展，便捷高效、覆盖城乡、更可持续的公共服务体系全面建成；共建共治共享的社会治理格局基本形成，社会化、法治化、智能化、专业化水平显著提高。

人民生活更加美好。全市现行标准下农村贫困人口全部脱贫，贫困县全部摘帽。城乡居民收入与地区生产总值同步增长，农村居民收入增速高于城镇居民，城乡居民收入差距进一步缩小。决胜全面小康目标如期实现，居民生活条件和生活质量全面改善，群众的获得感、幸福感、安全感更加充实、更有保障、更可持续。

美好蓝图需要实干来绘就，幸福生活要靠奋斗来创造。今后五年，新一届市政府将深入贯彻落实市第十一次党代会及历次全会精神，紧盯“四高一强一率先”奋斗目标，全面深化“9+2”工作布局、全力提升推进67个重大专项，着力抓好六个方面的主要任务，推动经济社会高质量发展。

——加快产业结构优化升级，全面深化“五强六新五特”现代产业体系，着力建设全国重要的先进制造业基地、现代服务业基地和国际文化旅游名城。坚持把产业转型作为高质量发展的根基，以供给侧结构性改革为主线，以“四双联动”为路径，以地企合作为平台，以实施现代产业体系11个重大专项为重点，深入推进重点产业转型发展行动计划，积极推进重点企业提质倍增，推动传统产业转型升级、新兴产业培育壮大，持续巩固“三产超二产”的产业结构。围绕加快六大产业园建设，做大做强先进制造业集群。全面加快高端装备、新能源汽车、智能制造、军民融合、轨道交通等重点产业园建设，积极创建“中国制造2025”国家级示范区、老工业基地和资源型城市产业转型升级示范区，让“洛阳智造”成为老工业基地的新名片。深入推进“企业上云”计划，组建工业互联网联盟。依托“一基地两园区”，加快建设国内有重要影响力的机器人及智能装备产业研发和生产基地。加快中原大数据云计算中心建设，打造中西部地区大数据产业与应用示范基地。围绕深化现代市场体系，支持民营经济发展壮大。全面提升推进4个重大专项，优化传统市场布局；积极培育发展新业态，持续推进“引金入洛”工程，深入实施电子商务产业转型攻坚行动。认真落实全省促进非公有制经济健康发展大会精神，把支持民营经济发展摆在更加突出位置，坚决破除各种隐性壁垒，制定完善市场准入负面清单，持续实施企业家能力提升、“小巨人”企业培育工程，加快形成大企业“顶天立地”、小企业“铺天盖地”的良好局面。围绕深化文化传承创新体系，推动文化旅游业“三个转变”。全面加快隋唐洛阳城国家历史文化公园建设，强力推进“一区、一轴、一带、两片、三环”等重要节点工程。加快建设华夏历史文明传承创新区，主动融入国家“大运河文化带”战略，突出特色，打造新时期历史文化保护利用的洛阳模式。全面提升推进5个重大专项，积极发展智慧旅游、全域旅游，实现文化旅游产业由“老三篇”向“新三篇”、“门票经济”向“产业经济”、“旅游城市”向“城市旅游”的转型升级。做好历史名城、名镇、名街、名村保护建设，确保二里头夏都遗址博物馆等标志性文化工程如期建成开放，博物馆、纪念馆总数达到100家，“博物馆之都”建设更加出彩。

——大力实施创新驱动发展战略，全面深化现代创新体系，着力建设国家创新型城市。始终把创新摆在发展全局的核心位置，突出抓好9个重大专项，加快“四个一批”建设，打造“双创”升级版，争创国家军民融合创新示范区，争做郑洛新国家自主创新示范区的领头羊。强化创新龙头带动。坚持把自创区作为创新驱动发展的核心载体，促进自创区、自贸区、高新区“三区”加快融合发展，引导“六组团”县市融入“三区”建设。持续推进创新主体创新平台“双倍增”行动，打造更多高水平的创新平台，集聚更多国内外高端创新资源，培育更多创新型领军企业。加速创新成果转化。深化科技体制机制改革，启动实施科技引领产业创新行动计划，着力突破一批关键核心技术，进一步打通“四个通道”、促进“四链融合”，推动“政产学研金用介”深度融合，让更多科研成果走出实验室、走进大市场、形成大产业。壮大创新人才队伍。人才是创新发展的第一资源。认真落实“1+4”人才工作政策，制定更有吸引力的激励措施，持续实施“河洛英才”“河洛工匠”计划，大力发展院士经济，精心育才、大力聚才、科学用才，为海内外人才施展抱负、成就梦想搭建广阔舞台，让河洛大地群英荟萃、人才辈出。

——实施更加积极主动的开放战略，全面深化现代开放体系，着力打造中西部地区对外开放新高地。坚持“引进来”与“走出去”相结合，提升推进8个重大专项，让洛阳开放的大门越开越宽广，形成全面开放新格局。加快“一带一路”主要节点城市建设，深化与沿线国家和地区的经济、文化联系，积极扩大国际产能合作和经贸合作，让更多的洛阳产品和洛阳企业走出去。全面提升开放平台优势，高水平建设自贸区，积极推进制度创新、政策落地，打造投资贸易便利、高端产业集聚、监管高效便捷、辐射带动作用突出的自由贸易园区；统筹抓好航空、铁路、公路等口岸平台建设，积极申建质检中心、标准化中心，争创国家服务外包示范城市。进一步放宽外资市场准入，围绕现代产业体系推进精准招商，大力引进一批技术水平高、带动作用强的龙头企业和重大项目。

——坚持以人的城镇化为核心，全面深化现代城镇体系、现代基础设施体系，着力建设幸福宜居的现代化城市。统筹做好规划建设管理“三篇文章”，协同推进提升现代城镇体系6个重大专项、现代基础设施体系10个重大专项，优化中心城区“三大板块”空间发展格局，持续深化“一中心六组团”城市发展战略，强化南部四县的生态保育和经济联系，着力完善“四级联动”城镇总体布局。坚持高水平规划。统筹编制好城市五期总规和各类专项规划，以精品意识做好城市设计，实现“多规合一”。积极推动“一中心六组团”产业承接、交通连接、生态对接，使每个组团都成为洛阳发展的重要支撑。坚持高标准建设。以绿“荫”城、以水“润”城、以文“化”城、以业“兴”城，以百城建设提质和文明城市创建为契机，统筹抓好旧城和新区、地上和地下基础设施建设，积极推进城市“双修”、地下综合管廊和“海绵城市”示范区建设，持续增强城镇综合承载能力。坚持精细化管理。积极推动互联网、大数据等信息技术在城市规划、建设、管理中的全方位应用，统筹抓好交通秩序治乱、市容卫生治脏、生态环境治污、城乡建设治违、公共服务治差，让城市运行更智能、人居环境更优美、群众生活更便利。

——牢固树立和践行绿色发展理念，全面深化生态环境建设体系，着力打造天蓝地绿水清的美丽洛阳。认真贯彻习近平生态文明思想和全国生态环境保护大会精神，像保护眼睛一样保护生态，像珍惜生命一样珍惜绿色，坚决打好污染防治攻坚战，全面提升推进7个重大专项，深入实施蓝天、碧水、净土、国土绿化、生态修复“五大行动”，努力实现“一河清水到洛阳”“清新空气到洛阳”“生态绿带连洛阳”，让好山好水好风光融入洛阳城乡各地。强化大气污染防治，积极推进中心城区通风廊道建设，下大力气优化能源结构、产业结构，实现优良天数逐年上升、污染天气逐年减少。全面落实河长制湖长制，深化“四河同治、三渠联动”综合治理，统筹推进山、水、林、田、城、园、路等综合治理和提升，早日实现“水清、岸绿、路畅、惠民”的目标。坚持“规划见绿、见缝插绿、提质优绿、协力植绿”，积极推进全域绿化，提高绿化档次和水平，加强小游园建设提升，打造“开窗见绿、出门进园、四季常绿、三季有花”的生态城市。

——坚持以人民为中心的发展思想，全面深化公共服务体系，着力增强群众的获得感、幸福感、安全感。民之所望、政之所向，洛阳人民对美好生活的向往就是政府工作的奋斗目标。加快构建公共服务体系，全面提升推进7个重大专项，让改革发展成果更多、更公平地惠及全市人民！坚持精准扶贫精准脱贫基本方略，打好“四大硬仗”，落实“六大行动”，推进“四大工程”，坚决打赢脱贫攻坚战。尽力而为、量力而行，集中力量做好普惠性、基础性、兜底性民生建设，织密织牢社会保障网，大力发展教育、医疗卫生、文化、体育等社会事业，全面提升公共服务供给质量。坚决打好防范化解重大风险攻坚战，着力防范化解各种重大风险隐患，守住不发生区域性、系统性风险的底线。牢固树立安全发展的理念，积极创建安全发展示范城市。健全突发事件预警应对体系，增强全社会防灾减灾救灾能力。完善立体化社会治安防控体系，深入开展“扫黑除恶”专项斗争，建立防范打击长效机制，始终保持惩治涉黑涉恶犯罪的高压态势，持续提升社会治理能力和水平，让洛阳人民安居乐业、社会大局安定有序。

三、当前重点工作

2018年是新一届政府的开局之年，全市经济社会发展的预期目标是：经济运行保持平稳，地区生产总值增长8.5%左右、实际工作中力争更好一些，全社会固定资产投资增长9%左右，社会消费品零售总额增长12%左右，进出口总值稳步增长；结构不断优化，规模以上工业增加值增长8.8%，“三二一”产业格局进一步巩固，先进制造业、现代服务业比重进一步提升，科技对经济增长的贡献率进一步提高，常住人口城镇化率提高1.8个百分点；质量效益持续提升，一般公共预算收入增长8.5%左右、税收占比不低于70%，节能减排、环境保护等约束性指标完成省下达任务；民生持续改善，实现10.65万农村贫困人口稳定脱贫，城镇新增就业10万人以上，调查失业率、城镇登记失业率分别控制在5.5%和4.5%以内，居民人均可支配收入与经济增长同步，居民消费价格指数控制在3%以内。

今年以来，全市上下团结拼搏、扎实工作，为完成全年目标奠定了坚实基础。下一步，我们将认真落实市委十一届七次全会暨重点工作推进会精神，着力抓好以下七个方面工作，持续推动经济社会高质量发展，为新一届政府工作开好局、起好步。

（一）着力推动实体经济健康发展。加快实施“三大改造”。建设12个智能车间和智能工厂，培育5个绿色工厂，打造绿色示范园区，确保年内全市40%的规模以上工业企业落实“三大改造”。以铝精深加工为重点，推动有色金属产业转型升级。持续深化地企合作。实施“两帮两促”进企业活动，拓展地企合作领域，推动今年签约的160个产业合作项目落地，确保开工率超90%。加强煤电油气运等生产要素综合协调，帮助企业解决运行中存在的问题。持续推进减税降费，确保全年降低企业各项成本30亿元以上。发展壮大现代服务业。培育1－2家省级跨境电子商务示范园区，启动中部电商港等项目，力争全年电子商务交易额超过1900亿元。加快华耀城商品市场、万邦国际物流园等重点商贸物流项目建设，建成河南（宜阳）医药产业园一期工程，改造提升宏进农副产品批发等2个市场，加快推进5个市场疏解外迁工作。抓好国家产融合作试点城市建设，做好华夏银行、浙商银行、渤海银行入驻服务工作。引导企业对接多层次资本市场，加大企业上市扶持力度，力争全年资本市场融资245亿元以上、新增贷款350亿元以上，银企洽谈会签约资金落实率达到85%以上。做优做强文化旅游业。积极推进隋唐大运河博物馆、二里头夏都遗址博物馆、隋唐洛阳城国家历史文化公园等项目建设，确保年内隋唐洛阳城应天门、“两坊一街”、南城墙一期项目主体完工。积极创建国家级旅游改革创新先行区。支持伏牛山旅游大联盟发展。谋划建设洛阳国际会展中心。精心筹备第37届牡丹文化节、第二届世界古都论坛。积极扩大有效投资。滚动实施合理有效投资行动计划，着力推进433个省市重点项目，突出抓好洛阳石化炼油结构调整一期、银隆新能源汽车、格力空调等重大项目，力争全年完成投资1030亿元。积极推进9个小微企业工业园建设，力争民营经济全年新上超千万元项目400个以上。围绕67个重大专项，提前谋划2019年重点项目，力争更多项目进入国家、省支持范围。积极防范化解金融等领域风险。坚持把防范化解金融风险与服务实体经济结合起来，依法严厉打击非法集资、金融犯罪，规范运作PPP项目，加强融资平台管理，积极防控政府债务风险。

（二）着力深化改革开放。持续深化供给侧结构性改革。严格执行去产能政策，统筹做好职工安置、债务处置等工作；有效化解房地产结构性库存，积极推进租售并举，促进房地产市场健康稳定发展。统筹推进重点领域改革。推动中铝洛铜等试点企业市场化法治化债转股；全面建立国有独资公司外部董事制度；继续推进脱钩企业改革，完成市属企业“三供一业”改造分离移交和16家子公司清理整合工作；支持服务驻洛央企省企深化改革、转型升级，确保驻洛央企“三供一业”改造分离移交工作全面完成。推动城投集团、旅发集团等向投资运营公司转型。高水平建设自贸区。全面落实160项改革试点任务，力争全年入驻企业1500家以上，引进资金100亿元以上；抓好金融科创平台建设，争取金融开放自主审批试点权限，吸引证券、期货等机构入驻自贸区。抓好跨境电商等平台建设，加快综合保税区申建工作。持续扩大对外开放。推动洛阳一类航空口岸扩大开放，推进洛阳机场公司化运营，确保全年旅客吞吐量达到130万人次。实施铁路货运通道“东联西进”计划，谋划建设多式联运中心、中欧班列甩挂点。加快推动布哈拉产业园建设，支持中信重工、中油一建、洛钼集团、省安装公司开展国际产能合作、实施国际项目。积极参加首届中国国际进口博览会、广州博览会、厦门国际投资贸易洽谈会等重大经贸活动，力争全年引进10亿元以上项目80个。推进与长安汽车、忠旺集团、京东集团、华为公司、省投资集团的合作项目早日落地。

（三）着力提升创新发展能力。加快自创区建设步伐。积极参与郑洛新·中关村双创基地建设，突出抓好工业CT、高铁轴承等一批创新引领型产业集群专项，推动解放军信息工程大学、郑州大学等在洛建设新型研发机构。大力实施“双倍增”行动。力争全年提升创新龙头企业20家，高新技术企业突破400家，科技型中小企业突破3500家。支持一拖集团、中航光电等企业申报国家级创新平台，推进国家光伏产品质量监督检验中心建设，引导鹏起实业等30家企业建设省级研发平台，新建创新平台280个。持续推动大众创业万众创新。加快建设中信重工等“双创”示范基地，打造语音技术专业化众创空间等20个“双创”载体，省级创业孵化示范基地达到7家。办好第七届中国创新创业大赛先进制造行业总决赛、直通硅谷创新创业大赛，巩固提升“院士洛阳行”活动合作成果。促进军民深度融合发展。加快运营500亿元军民融合产业投资基金，争取国家国防科技工业军民融合产业投资基金子基金落户洛阳。建设洛阳军民融合创新研究院、军民融合产业园，支持中信重工建设先进装备及军民融合产业创新科技园，推动与北京航空航天大学、中国兵器装备集团等重大合作项目尽快落地。推动科研成果加快转化。高效运营洛阳科技金融大市场，建设提升20家科技服务业示范机构，引导商业银行设立科技支行，启动运营天使、种子投资引导资金，“科技贷”合作银行超过6家。支持河南科技大学牵头建设河南高端轴承研究院，支持企业组建省级产业技术创新战略联盟。鼓励、支持科研院所帮助企业开发新产品、新技术，增强中小微企业市场竞争力。

（四）着力提高城镇综合承载能力。提升中心城区功能品质。加快地铁1、2号线建设，力争金谷园北路等工程10月底前建成通车，王城大道快速化直线段2019年10月底前主体通车。加快推进西环路、北环路、瀍涧大道建设，完善“井字加外环”城市快速路网，年底前完成20条重点道路、50条背街小巷提升改造任务。积极创建公交都市，确保308辆新能源公交车年底前全部投用。加快国家产城融合示范区建设，提高伊滨经开区规划建设水平。加强基础能力建设。加快二广高速市区段改扩建、洛济等高速建设，确保尧栾西高速如期竣工，推动新伊、渑栾、栾卢高速实质性开工。抓好伊川－偃师等3条组团高速、洛宁－嵩县－汝州生态环线高速前期谋划工作。改造提升洛伊、洛偃、洛新、洛吉等快速通道。推动洛阳与豫西北各市交通基础设施对接联通。协调推进洛阳机场综合枢纽和洛宁、栾川通用航空机场前期工作，加快推进万安山通用航空机场建设。加快三洋铁路洛阳段、龙门综合交通枢纽建设，确保龙门长途客运南站10月底试运行。做好呼南高铁豫西通道、焦柳铁路洛阳段改线和洛宜支线外迁等项目前期工作。加快洛宁抽水蓄能电站建设。集中开工110千伏及以上电网建设项目14项。新增供热面积600万平方米，力争中心城区集中供热率提高到80%。做大做强县域经济。坚持百城建设提质与文明城市创建相结合，持续提高县域城镇规划建设水平，抓好482个重点项目，确保270个竣工投用。完成洛济、洛汝、洛渑、洛巩等豫西北特色产业带规划。

（五）着力实施乡村振兴战略。扎实推进脱贫攻坚。做好1个深度贫困县和206个深度贫困村脱贫工作，确保10.65万贫困人口稳定脱贫，栾川、洛宁、宜阳、伊川4个贫困县脱贫摘帽，完成20497人易地扶贫搬迁任务。积极发展现代农业。加快高标准农田建设和农机装备现代化，确保粮食总产稳定在40亿斤以上。培育壮大优质杂粮、果蔬、牡丹等特色高效农业，实施特色农业“百乡百村”示范创建工程，抓好23个沟域经济示范区建设，扶大扶强10个农业产业化集群。持续深化农村改革。完善承包地“三权分置”制度，落实好第二轮土地承包到期后再延长30年政策，推进农村房屋不动产登记颁证。深化农村集体产权制度改革，抓好水务、林业、粮食、供销等重点改革。完善农村基础设施。发展节水灌溉面积2万亩，新增有效灌溉面积2万亩。力争小浪底南岸灌区年内开工建设，前坪水库1.4万人移民搬迁安置工作大头落地，改善285个贫困村饮水条件，年底前再建成330公里农村公路。加快建设美丽乡村。实施农村人居环境整治三年行动和美丽乡村创建达标行动，加强农村生活垃圾和污水治理，推进农村“厕所革命”。培育9个中心镇、6个特色镇、50个美丽乡村示范点，完成154个村庄环境综合整治。

（六）着力打好污染防治攻坚战。坚决打赢蓝天保卫战。认真落实蓝天保卫战三年行动计划，推进汾渭平原区域综合治理，强化“六控”措施，持续抓好“散乱污”企业整治，加强污染天气应急管控和绿色调度。大力推进清洁取暖试点城市建设，统筹抓好集中供热、建筑节能改造、“双替代”等工作。加大水污染防治力度。深入实施“水十条”，全面推进水源地污染综合防治，深化“四河三渠”综合治理，确保完成洛河西段河道治理、洛河东段和伊河东段绿化、伊水游园提升等年度任务，瀍河、涧河治理全面开工，基本完成县城和18个产业集聚区黑臭水体截污纳管，全部消除城市建成区黑臭水体，地表水和饮用水水源地水质全面达标。扎实推进土壤污染防治。全面实施“土十条”，推进土壤污染综合防治先行区建设。实施农用地土壤环境分类管理和建设用地准入管理，加强生活污染源管控，抓好农村农业面源污染防治，严格危险废物环境监管、涉重金属企业风险管控。强化生态保护修复。实施国土绿化和生态修复行动，推动绿色矿山建设，11月底前完成18座尾矿库综合治理任务，提高尾矿库资源化利用率。加快小游园、嵩县陆浑湖和伊川伊河国家湿地公园试点建设，完成周山森林公园核心区提升工程。

（七）着力保障和改善民生。认真办好人民满意教育。深入实施贫困地区义务教育“全面改薄”和扩充城镇义务教育资源五年计划，着力缓解“择校热”“大班额”等突出问题。办好学前教育，加大普惠性幼儿园、乡村幼儿园建设力度，新建、改扩建公办幼儿园76所、农村义务教育寄宿制学校114所，招聘特岗教师1225名。大力实施高中教育普及攻坚计划和第二期特殊教育提升计划。加快河南中医药大学洛阳平乐正骨学院和洛阳职业技术学院新校区建设，推进职业教育产教融合。支持和规范民办教育发展。支持在洛高校发展，鼓励与知名高校共建特色学院。持续提高社会保障水平。城乡居民基本医保财政补助提高到每人每年490元。做好异地就医直接结算、困难群众医疗救助工作，提高农村贫困人口门诊慢性病和重特大疾病保障水平。加大棚户区改造力度，确保全年开工安置房14449套，基本建成29475套。加快实施健康养老“十百千”示范项目，建成医养结合智慧型社区养老服务中心30个、农村幸福院200个，增加养老床位3000张。健全农村留守儿童、妇女、老年人关爱服务体系，全面完成150个基层“儿童之家”建设任务。完成3888户建档立卡贫困重度残疾人家庭无障碍改造。加大重度失能残疾人集中托养中心建设力度，提高入住率。扎实推进健康洛阳建设。抓好国家中医骨伤区域医疗中心、中医药传承创新等重大项目。进一步做好分级诊疗工作，完善城市公立医院布局，积极推进伊滨医院、儿童医院项目建设。做好城市医联体、县域医共体试点工作。加快县级医院、乡镇卫生院改扩建和150个村卫生室建设提升，加强乡村医生队伍建设，提高基层医疗卫生服务能力。积极创建国家全民运动健身模范市，改造建设西工体育场、自行车赛场、文博体育公园，打造15分钟健身圈。全面启动十四届省运会筹备工作。积极做好国家卫生城市复审迎检工作。大力促进文化繁荣发展。深入推进群众性精神文明创建，提升市民文明素养。加快市中心图书馆、非遗展示馆、文化馆、少儿图书馆、方志馆等公共文化设施建设，建成城市书房100座，所有乡镇（街道）和行政村（社区）建成基层综合性文化服务中心。加强网络建设和治理，营造健康清朗网络环境。筑牢社会安全稳定底线。严格落实安全生产责任制，强化重点行业领域安全专项治理，持续保持“一杜绝、三下降”。加强食品药品安全监管，让人民群众吃得更安全、更放心。积极推动社会治理创新，依法妥善解决群众合理诉求。提升社区物业管理水平。扎实开展“扫黑除恶”专项斗争，聚焦“六个围绕”，紧盯“四净一促”目标，坚持“四结合、四同步”，坚决“亮剑”、重拳出击，让黑恶势力在洛阳无所遁形。全面加强国防教育，努力提高国防动员和后备力量建设质量。大力支持驻洛部队建设，争创全国双拥模范城“八连冠”。

四、加快建设人民满意的政府

我们将不忘初心、牢记使命，勇于担当、勤勉履职，全面加强政府自身建设，以党的建设高质量推动经济发展高质量。一是坚持忠诚担当，加强政治建设。牢固树立“四个意识”，坚定“四个自信”，自觉践行“两个坚决维护”，始终在思想上政治上行动上同以习近平同志为核心的党中央保持高度一致，认真落实党中央国务院、省委省政府和市委决策部署，持续抓好中央、省委巡视反馈意见整改工作，确保听令而行、闻令而动、政令畅通。二是转变政府职能，优化发展环境。加快推进政府机构改革，持续优化职能配置。紧盯群众办事、企业发展和政务服务中的痛点、堵点、烦心事，深化“放管服”改革，推进“一网、一门、一次”改革，加快“市民之家”智能化建设，打造全市政务大数据平台，提高政务服务便利化水平。推行清单管理，加强“双随机一公开”监管，依法维护企业利益，构建“亲”“清”政商关系，打造中西部地区最佳营商环境。加强政务诚信建设，打造诚信洛阳。三是坚持依法行政，规范权力运行。加快法治政府建设，加强重点领域立法，严格履行重大行政决策法定程序，促进严格规范公正文明执法，坚决杜绝以言代法、以权压法、徇私枉法。严格执行人大及其常委会决定，提高人大议案、代表建议和政协提案办理质量。依法接受人大及其常委会法律监督、工作监督和政协民主监督，认真听取民主党派、工商联、无党派人士和各人民团体意见，主动接受社会监督和舆论监督，全面深化政务公开，让行政权力始终在阳光下运行！四是狠抓工作落实，提升行政效能。加强政府系统学习培训，弘扬焦裕禄同志的“三股劲”，提升素质能力，克服“本领恐慌”。大力倡树“清新简约、务本责实”的作风导向，摒弃繁文缛节，杜绝形式主义，严肃整治懒政怠政行为，谋实事、出实招、求实效，集中精力抓好改革发展稳定具体工作。勇于争先创优，以“钉钉子”精神攻坚克难，抢时间、提速度，以高效率保障高标准。五是持续正风肃纪，保持清廉本色。扛实抓牢全面从严治党主体责任，认真落实党风廉政建设“一岗双责”，深入贯彻中央八项规定及其实施细则精神，驰而不息纠正“四风”。加强财政监管和审计监督。严厉查处各类违纪违法案件，坚决整治侵害群众利益的不正之风和腐败问题。

各位代表！新时代开启新征程，新使命呼唤新作为。让我们更加紧密地团结在以习近平同志为核心的党中央周围，高举习近平新时代中国特色社会主义思想伟大旗帜，在市委的坚强领导下，在市人大、市政协的监督支持下，锐意进取，务实重干，为实现“四高一强一率先”奋斗目标、开启新时代洛阳全面建设现代化新征程、在中原更加出彩中谱写浓墨重彩的洛阳篇章做出新的更大的贡献！
